# Pickles and ice cream! Food cravings in pregnancy: hypotheses, preliminary evidence, and directions for future research

**DOI:** 10.3389/fpsyg.2014.01076

**Published:** 2014-09-23

**Authors:** Natalia C. Orloff, Julia M. Hormes

**Affiliations:** Health Behaviors Laboratory, Department of Psychology, University at Albany – State University of New YorkAlbany, NY, USA

**Keywords:** pregnancy, craving, restraint, eating disorders, food, chocolate, perimenstrual

## Abstract

Women in the United States experience an increase in food cravings at two specific times during their life, (1) perimenstrually and (2) prenatally. The prevalence of excess gestational weight gain (GWG) is a growing concern due to its association with adverse health outcomes in both mothers and children. To the extent that prenatal food cravings may be a determinant of energy intake in pregnancy, a better understanding of craving etiology could be crucial in addressing the issue of excessive GWG. This paper reviews the available literature to corroborate and/or dispute some of the most commonly accepted hypotheses regarding the causes of food cravings during pregnancy, including a role of (1) hormonal changes, (2) nutritional deficits, (3) pharmacologically active ingredients in the desired foods, and (4) cultural and psychosocial factors. An existing model of perimenstrual chocolate craving etiology serves to structure the discussion of these hypotheses. The main hypotheses discussed receive little support, with the notable exception of a postulated role of cultural and psychosocial factors. The presence of cravings during pregnancy is a common phenomenon across different cultures, but the types of foods desired and the adverse impact of cravings on health may be culture-specific. Various psychosocial factors appear to correlate with excess GWG, including the presence of restrained eating. Findings strongly suggest that more research be conducted in this area. We propose that future investigations fall into one of the four following categories: (1) validation of food craving and eating-related measures specifically in pregnant populations, (2) use of ecological momentary assessment to obtain real time data on cravings during pregnancy, (3) implementation of longitudinal studies to address causality between eating disorder symptoms, food cravings, and GWG, and (4) development of interventions to ensure proper prenatal nutrition and prevent excess GWG.

## OVERVIEW

Food cravings are a common phenomenon, especially in women in the United States (U.S.), and have been implicated in a range of weight- and eating-related pathology. Cravings in women have been shown to increase in frequency and intensity at two distinct times: during the perimenstrum (i.e., a period of about eight days around the onset of menstruation) and in pregnancy. Perimenstrual cravings for chocolate have been the focus of significant attention from researchers in recent years, resulting in enhanced insight into the mechanisms underlying craving etiology. Cravings in pregnancy, on the other hand, remain relatively understudied. This gap in the literature is especially striking given the steady rise in prevalence of excess gestational weight gain (GWG) during the end of the last century, which is related to adverse health outcomes in mothers and their children, along with a growing understanding of the causal role of food cravings in the etiology of overweight and obesity. Thus, a call for a renewed focus on research in this area is warranted.

This paper seeks to highlight the importance of gaining a better understanding of the mechanisms underlying food cravings as a potentially modifiable determinant of energy intake and nutritional quality in pregnancy. We will begin with a brief introduction to food cravings both in general and specifically in pregnancy, followed by an overview of the adverse health effects of excess GWG. We will then introduce a theoretical framework of craving etiology that integrates key results from work on perimenstrual chocolate craving and argue that this framework can serve as a useful blueprint for the study of food cravings in pregnancy. We will review major hypotheses regarding craving etiology and examine the extent to which they are supported or refuted by the existing literature on prenatal eating behaviors. We will conclude with thoughts on future directions for research in this area. It is important to note that an exhaustive review of the literature in this field is beyond the scope of the present paper. Instead, we aim to call attention to the importance of studying food cravings in pregnancy in so far as they may be implicated in the growing rates of gestational overweight and obesity and associated adverse health effects in U.S. mothers and their children. Our primary goal is to take advantage of the knowledge gained from the study of cravings in other domains to formulate testable hypotheses about the underlying causes of food cravings in pregnancy.

## FOOD CRAVINGS: AN INTRODUCTION

Food cravings are strong urges for foods that are more specific than mere hunger and very difficult to resist (Gendall et al., 1997; Pelchat, 2002; Hormes and Rozin, 2010). Food cravings are a common phenomenon, at least in some areas of the world. Between 68 and 97% of college-aged men and women in North America report ever having experienced a craving for a specific type of food (Weingarten and Elston, 1990; Zellner et al., 1999). It is tempting to think of food cravings as far less harmful than strong urges for alcohol, tobacco, and other drugs, which are known to trigger relapse and interfere with successful abstinence from substance use (Bottlender and Soyka, 2004; Sinha et al., 2006; Ferguson and Shiffman, 2009; Evren et al., 2012). However, a growing body of research now points to a significant role of food cravings in the development and maintenance of eating- and weight-related pathology, including overweight, obesity, bulimia nervosa, binge eating disorder, and failure to sustain weight losses (Gendall et al., 1997; Lafay et al., 2001; Lowe, 2003; Lowe and Levine, 2005; Forman et al., 2007; Vander Wal et al., 2007). For example, food cravings have been identified not only as reliable predictors of subsequent consumption of the desired food (Forman et al., 2007), but also as potential triggers for episodes of binge eating, especially in bulimic and overweight individuals (Bjoervell et al., 1985; Kales, 1990). In spite of a steadily growing number of studies in this field, the exact mechanisms underlying the etiology of food cravings have yet to be elucidated. There has been a recent increase in efforts to develop interventions targeting food cravings and studies have tested the efficacy of diverse approaches, including brief guided imagery (Hamilton et al., 2013), use of self-help manuals (Rodriguez-Martin et al., 2013), acceptance based strategies (Forman et al., 2007; Alberts et al., 2010), and biofeedback (Meule et al., 2012) in preventing or reducing food cravings. It should be noted that most of these interventions were developed specifically for individuals who identify as strong cravers (Meule et al., 2012), non-clinical populations (Forman et al., 2007; Hamilton et al., 2013), or those enrolled in weight loss trials (Batra et al., 2013). More work to test the efficacy of these interventions specifically in clinical populations is warranted.

The prevalence and nature of food cravings varies significantly depending on the geographic region under investigation (Hormes and Rozin, 2010; Hormes, 2014). Food cravings seem to be most commonly reported by individuals in North America and chocolate has consistently been found to be the most commonly craved food in the U.S. (Rozin et al., 1991; Osman and Sobal, 2006). Within the U.S., the type, frequency, and intensity of reported food cravings vary markedly by demographic characteristics. Younger individuals are more likely to experience food cravings, with prevalence decreasing steadily with age (Pelchat, 1997). Women primarily report strong urges to consume sweets, while men typically crave savory foods, especially when stressed (Zellner et al., 1999, 2007). Women in the U.S. are twice as likely to experience cravings for chocolate as compared to men. This difference in prevalence appears attributable, primarily, to a pronounced increase in chocolate craving frequency and intensity during the perimenstrum, an eight days period beginning about four days prior to the onset of menstruation, for around half of female cravers (Rozin et al., 1991; Zellner et al., 2004; Hormes and Rozin, 2009). In addition to the characteristic perimenstrual rise in chocolate craving, it appears that many U.S. women may also experience an increase in food cravings during pregnancy (Pope et al., 1992). In spite of a growing interest in the study of mechanisms involved in the etiology of cravings in other domains, food cravings in pregnancy are poorly understood and widespread speculation about their meaning and significance by laypersons and the media stands in stark contrast to a lack of empirical research on the subject.

## FOOD CRAVINGS IN PREGNANCY

An estimated 50–90% of U.S. women experience cravings for specific foods during pregnancy (Worthington-Roberts et al., 1989; Pope et al., 1992). Very few women report food cravings exclusively during pregnancy; most have a history of pregravid cravings for a variety of substances (Gendall et al., 1997). In terms of temporal patterns, it has been reported that food cravings typically emerge by the end of the first trimester. For example, among a sample of 400 white adult women 76% reported craving at least one food item by the 13th week of pregnancy (Tierson et al., 1985). The most common trajectory of food cravings across gestation shows a peak in frequency and intensity during the second trimester, followed by a subsequent decline as the pregnancy progresses to term (Pope et al., 1992; Bayley et al., 2002; Belzer et al., 2010). Research has also consistently documented a significant drop in cravings following delivery (Worthington-Roberts et al., 1989; Belzer et al., 2010).

A 1978 study retrospectively examined prevalence and types of cravings in a group of 250 pregnant women and demonstrated that the most commonly craved items included sweets (i.e., ice cream and candy), dairy, starchy carbohydrates, fruits, vegetables, and fast food (Hook, 1978). A 1992 survey of pregnant adolescents found frequent reports of cravings for sweets, fruits, fast foods, pickles, ice cream, and pizza (Pope et al., 1992). More recent studies showed similar cravings, with women endorsing a desire for fruit juice, fruit, sweets, desserts, dairy, and chocolate (Flaxman and Sherman, 2000; King, 2000). Prenatal cravings for salty or savory foods are somewhat less commonly reported (Hook, 1978; Pope et al., 1992; Bayley et al., 2002), with the notable exception of women who experience cravings exclusively during pregnancy (Gendall et al., 1997). This subset of women were found to endorse cravings for savory, rather than sweet foods (Gendall et al., 1997). Given the lack of current data on the nature of food cravings in pregnancy we recently conducted a small pilot study examining women’s posts on pregnancy-related blog websites about the topic of food cravings^1^. Among 200 posts surveyed, the most commonly reported cravings were for sweets, calorically dense savory carbohydrates like pizza or chips, animal proteins, and fruits (**Table 1**). Prior research also points to certain temporal patterns in the types of foods craved over the course of pregnancy. Cravings for savory substances appear to be strongest during the first trimester, with a tapering of urges during the later stages of peripartum (Belzer et al., 2010). In a large number of women, a preference for sweet foods reaches peak intensity during the second trimester (Bowen, 1992). Urges for salty substances tend to emerge later on in pregnancy, with preference for and intake of salty foods increasing in the later stages of gestation in both pregnant adults (Bowen, 1992; Crystal et al., 1999) and teens (Skinner et al., 1998).

**Table 1:** Most common cravings (overlapping %) reported by pregnant women (*n* = 200) posting on popular pregnancy blogs.

Rank	Substance Craved	%
1	Sweets (e.g., chocolate, candy)	25.9
2	Carbohydrates, high-calorie, savory (e.g., pizza, chips)	19.3
2	Animal protein (e.g., steak, chicken)	19.3
4	Fruit	18.8
5	Dairy, high-calorie, savory (e.g., cheese, sour cream)	17.8
5	Carbohydrates, other (e.g., pretzels, cereal)	17.8
7	Fast food (e.g., Chinese, Mexican, falafel)	17.3
8	Cold foods (e.g., ice cream, slurpee)	13.2
9	Vegetables	12.2
10	Dairy, high-calorie, sweet (e.g., ice cream, milkshakes)	11.7

It is important to distinguish food cravings in pregnancy from pica, a condition characterized by (1) persistent eating of non-nutritive substances such as soils and clay (geophagia), ice (pagophagia), and laundry or corn starch (amylophagia; Anderson, 2001; Corbett et al., 2003) for a period of at least one month, (2) consumption of non-nutritive substances in a manner that is inappropriate to the developmental level of the individual, and (3) eating of non-nutritive substances that is not part of a culturally supported or socially normative practice (American Psychiatric Association, 2013). The presence of pica is not exclusive to pregnant women and the condition can be diagnosed in non-pregnant individuals of all ages. A number of theories attempting to explain the etiology of pica have been discussed in detail elsewhere (Young, 2010) and typically implicate factors such as nutritional deficiencies, a preference for the taste, smell, or texture of the craved substances (Cooksey, 1995), or the consumption of non-food items as a coping mechanism to relieve stress (Mills, 2007). Estimates of prevalence of pica in the U.S. vary widely. In our convenience sample of women posting on pregnancy-related websites in the U.S. only 3.0% (*n* = 6) indicated strong urges for non-food substances, which is consistent with an early study citing prevalence rates of pica around 1.6% (Hook, 1978). However, since then it has been reported that as many as one fifth of women who are considered as having a high-risk pregnancy endorse pica behaviors (Mills, 2007). Pica in pregnancy is more common in certain demographic groups, with relatively higher prevalence in African–Americans, immigrants to the U.S., women living in rural areas, and those that have a family history of pica (Horner et al., 1991; Thihalolipavan et al., 2013). Of note, the practice of consuming non-nutritive substances is thought to be present in a number of different cultures across the world (Geissler et al., 1999) and the consumption of non-nutritive substances as part of culture-specific practices has been observed in countries like Kenya where pregnant women were found to eat clay on a regular basis because of beliefs about its impact on fertility and reproduction (Geissler et al., 1999).

## ADVERSE HEALTH EFFECTS OF EXCESS GESTATIONAL WEIGHT GAIN

Food intake in pregnancy has been the focus of increasing attention from researchers, health care providers, and policy makers alike due in part to a growing awareness of the rising prevalence and significant adverse consequences of excess GWG for the health of both mothers and their children. The Institute of Medicine (IOM) defines excess GWG in singleton pregnancies as 35+ pounds in women of normal pre-pregnancy weight, 25+ pounds in overweight women, and 20+ pounds in women who are obese (Rasmussen and Yaktine, 2009). While there are multiple components of GWG, including the weight of the fetus, placenta, and amniotic fluid, much of the variance in weight gained in pregnancy is accounted for by an increase in fat mass (Kaiser et al., 2008; Rasmussen and Yaktine, 2009). Despite efforts to combat obesity in the U.S., the prevalence of excess GWG is on the rise: according to the National Research Council (NRC) and the IOM there was a 20–25% increase in U.S. women gaining more than 40 pounds during pregnancy from 1990 to 2003 (National Research Council and Institute of Medicine, 2007), and around half of all pregnancies currently result in GWG that exceeds IOM guidelines (Oken et al., 2007; Wrotniak et al., 2008; Chu et al., 2009; Rasmussen and Yaktine, 2009).

While maternal underweight and insufficient GWG have long been known to have serious adverse effects on the health and growth of the fetus (Ehrenberg et al., 2003; Han et al., 2011), excessive gestational weight is emerging as a potentially even greater threat to the health and wellbeing of both women and children (Kaiser et al., 2002, 2008). Excess GWG has been linked to a number of adverse short- and long-term health outcomes in mothers and their offspring (Cox and Phelan, 2008), and excess weight is currently among the most common high-risk obstetric conditions (Galtier-Dereure et al., 2000). Overweight and obesity are linked to higher rates of cesarean sections and greater cost of obstetric care (Galtier-Dereure et al., 2000; Stotland et al., 2004; Vahratian et al., 2005). Additional complications associated with excess GWG have been described in detail (Rasmussen and Yaktine, 2009) and include increased risk of gestational diabetes, hypertension, preeclampsia, delivery complications, perinatal fatality, neural tube defects, neonatal hypoglycemia, and failure to initiate breastfeeding (Hilson et al., 1997, 2006; Galtier-Dereure et al., 2000; Kaiser et al., 2002, 2008; Thorsdottir et al., 2002).

By following guidelines for GWG women may be able to avoid excessive postpartum weight retention, which results in greater short- and long-term risk of maternal overweight and obesity (Rooney and Schauberger, 2002; Linne et al., 2004; Rooney et al., 2005; Amorim et al., 2007; Nohr et al., 2008). Interestingly, data suggest that the correlation between inadequate GWG and poor fetal growth is weaker than the relationship between excess weight gain in pregnancy and maternal postpartum weight retention (Scholl et al., 1995; Kaiser et al., 2002). Excess GWG is also a strong predictor of macrosomia (Stotland et al., 2004) and overweight/obesity in children and adolescents (Oken et al., 2007, 2010; Wrotniak et al., 2008), highlighting the potential impact of excess weight gain in pregnancy on risk for weight-related pathology across the lifespan.

Prior research has sought to identify risk factors for excessive weight gain in pregnant women. A range of physiological variables, such as insulin sensitivity and basal metabolic rate, have been hypothesized to influence GWG (Rasmussen and Yaktine, 2009). Environmental context, including lack of access to physical spaces for exercise (Laraia et al., 2007), and sociodemographic variables, such as race/ethnicity (Chu et al., 2009), higher levels of education (Chu et al., 2009), younger age (Howie et al., 2003), and food insecurity (Olson and Strawderman, 2008), have been shown to be at least weakly correlated with an increased risk for excess GWG. For example, white women in the U.S. gain on average 2.0 kg more than their African–American counterparts, which is an increase from a survey conducted at the same hospital three decades prior that showed only a 0.9 kg difference between the two (Eastman and Jackson, 1968; Caulfield et al., 1996). Psychosocial factors including depression, anxiety, stress, internal locus of control, and self-esteem have all been found to correlate with excess GWG (Abraham et al., 1994; Clark and Ogden, 1999; Gee and Troop, 2003; Hill et al., 2013).

There is also preliminary evidence to suggest that food cravings may be an important determinant of prenatal energy intake and risk factor for excess GWG. This assertion is supported in part by a recent study which found that cravings during pregnancy were the only significant predictor of excess GWG in a sample of overweight African–American women (Allison et al., 2012). As noted earlier, food cravings are known to lead to an increase in consumption of the desired foods in both the general and certain clinical populations. Research points to a similar effect of food cravings in pregnancy: cravings for sweets, desserts, and chocolates have been shown to result in a general increase in consumption of sugary foods and beverages and overall caloric intake in pregnant women (Tierson et al., 1985; Pope et al., 1992; Belzer et al., 2010). In order to be able to target food cravings as a means of preventing or minimizing excess GWG, a better understanding of the mechanisms underlying strong urges for specific foods specifically during pregnancy is critical.

Popular hypotheses regarding the causes of food cravings in pregnancy implicate hormonal fluctuations, changes in sensory perception, maternal and/or fetal nutritional needs and preferences, adaptive mechanisms protecting the fetus from toxins, cultural norms and practices, and cognitive or affective characteristics of the pregnant woman (King, 2000; Patil, 2012). In the small pilot study mentioned earlier we sought to gather qualitative information about pregnant women’s own beliefs about the meaning and significance of their food cravings. Of the women who posted to the blog websites surveyed, 16.2% (*n* = 32) mentioned a perceived cause for their cravings. Responses varied widely but the more commonly cited hypotheses aligned closely with the existing literature: women speculated about cravings as a reaction to food restrictions (either self-imposed or prescribed by a physician, *n* = 6) or nutritional deficits (*n* = 5). Some postulated that cravings were indicative of the gender of the child (*n* = 5) or reflective of the parents’ or fetus’ food preferences (*n* = 3). A few women thought their cravings were largely due to external cues or triggers (*n* = 3) while others saw them as a byproduct of gestational diabetes (*n* = 2), a response to thirst (*n* = 2), a reaction to nausea or morning sickness (*n* = 2), or the result of changes in taste perception brought on by pregnancy (*n* = 2).

## PERIMENSTRUAL CHOCOLATE CRAVING: A BLUEPRINT

In examining popular hypotheses regarding craving in pregnancy their close resemblance to the theorized causes of perimenstrual chocolate craving is striking. Chocolate, by far the most commonly craved food in the U.S., is unique in many ways: it has a very recognizable smell, high caloric density, and distinctive melt-in-your-mouth feel (Rozin et al., 1991; Hormes, 2014). The characteristic pattern of cyclically fluctuating chocolate craving in many U.S. women described earlier has motivated a body of research specifically examining perimenstrual chocolate craving. Major findings from this work have previously been summarized in some detail elsewhere (Hormes, 2014). Accounts regarding the etiology of perimenstrual chocolate craving can be categorized as focusing on biochemical/physiological versus contextual/psychosocial mechanisms. Popular hypotheses attribute craving to cyclic fluctuations in levels of ovarian hormones, pre- and perimenstrual nutritional deficits, and pharmacologically active ingredients in chocolate that serve to alleviate symptoms that arise specifically around the onset of menstruation. More recently, research has shifted toward exploring the role of cultural and psychosocial factors in the emergence of perimenstrual chocolate craving. These parallels suggest that existing research on the causes of perimenstrual chocolate craving may serve as a sort of blueprint for the study of cravings in pregnancy.

**FIGURE 1:**
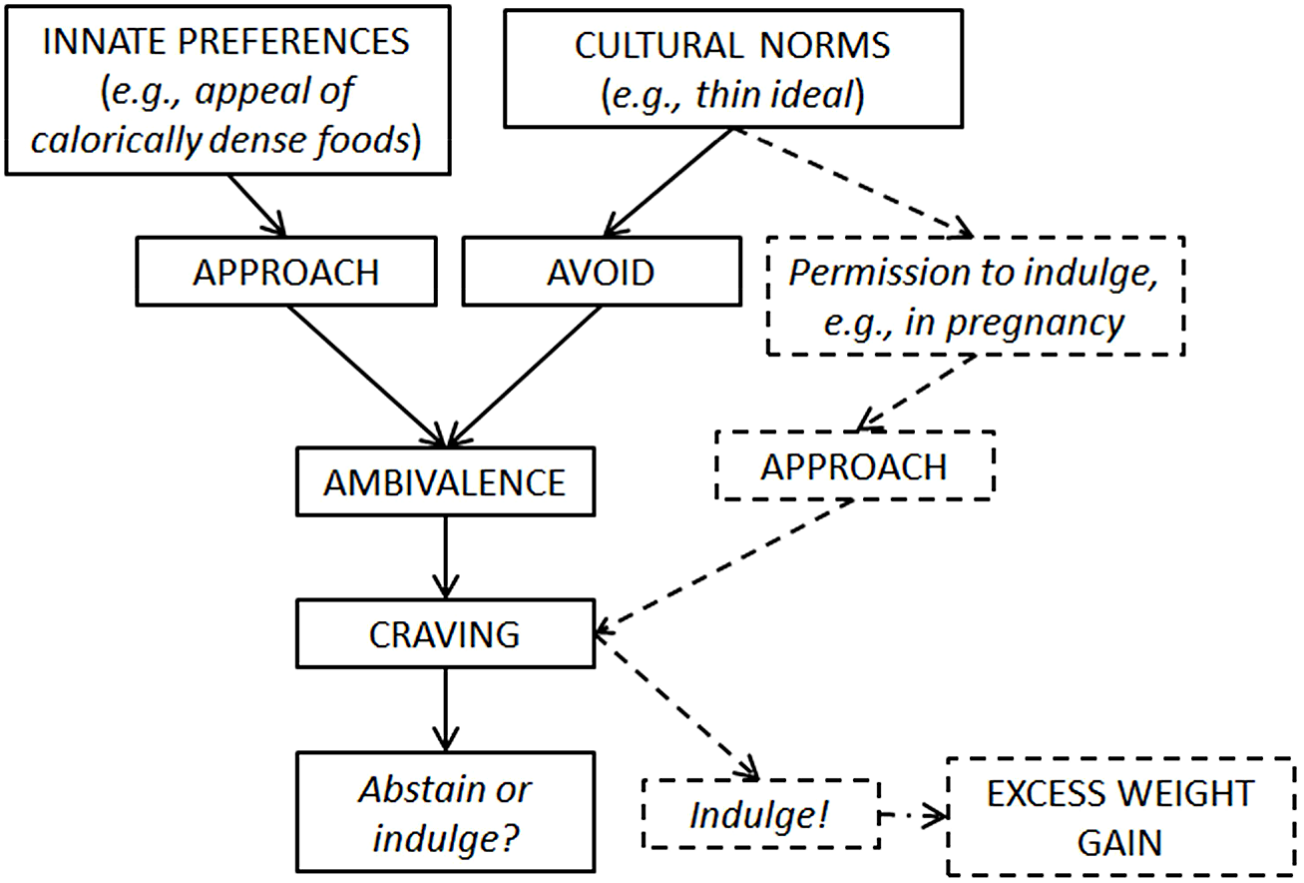
**Proposed model of craving etiology.** Craving is hypothesized to be due to competing approach-avoidance conflicts brought about by exposure to foods that are perceived as being simultaneously appealing (due to an innate preference for high-calorie, sweet, and fatty foods) and forbidden (due to cultural norms prescribing restrained intake and a thin figure). While most individuals are thought to attempt to resolve the resulting ambivalence in favor of abstinence (represented by the solid lines), pregnancy is hypothesized to be a culturally sanctioned permissive factor, allowing women to circumvent their usual conflicting response and efforts to restrict intake and indulge in foods that they would otherwise avoid, resulting in increased intake and heightened risk for weight gain specifically during pregnancy (represented by the dashed lines).

We have previously proposed a model that integrates findings regarding the role of contextual and psychosocial factors in craving etiology and provides a conceptual framework for the study of cravings across multiple domains, including food cravings in pregnancy (**Figure 1**; Hormes, 2014). The model postulates that craving results from ambivalence or a tension between approach (i.e., the desire to indulge) and avoidance (i.e., efforts to restrict consumption) tendencies toward highly palatable foods. It is assumed that most individuals – and U.S. women in particular – seek to resolve this ambivalence in favor of abstinence, thereby *de facto* increasing the likelihood that they will crave the avoided food due to an enhanced salience of relevant cues. The model furthermore proposes that certain culturally defined cues signal occasional permission to break restraint, resulting in episodic consumption (and, potentially, overconsumption) of craved foods. It is hypothesized that in the U.S., both the perimenstrum (“PMS”) and pregnancy act as such culturally sanctioned disinhibitors, resulting in the characteristic patterns of increased craving frequency and intensity (and, as a result, consumption) specifically at these times. In other words, contrary to previous models of craving etiology, our model does not consider the perimenstrum and pregnancy a direct *cause* of cravings, but instead views them as a *catalyst* or permissive factor, allowing women to acknowledge and give in to otherwise unacceptable desires for highly palatable foods.

In the remainder of this paper we will examine major hypotheses regarding craving etiology in more detail, starting with a discussion of the role of hormonal, nutritional, and pharmacological factors, followed by an overview of evidence in favor of a role of cultural and psychosocial variables. We will review findings from research on perimenstrual chocolate craving and explore the extent to which the literature on eating behaviors in pregnancy supports or refutes translation of the proposed theoretical model into the domain of food cravings in pregnancy. We will attempt to point to gaps in the literature and outline directions for future research, keeping in mind the ultimate goal of identifying targets for interventions to reduce the prevalence of excess GWG and associated adverse health effects.

## HYPOTHESIS 1: CRAVING IS CAUSED BY FLUCTUATING LEVELS OF HORMONES

Given the cyclic nature of perimenstrual chocolate craving, early hypotheses implicated fluctuations in the ovarian hormones estrogen and progesterone in craving etiology. Though initially quite compelling, there is generally a lack of empirical support in favor of this view. Levels of hormones involved in regulating the menstrual cycle are not significantly correlated with changes in the frequency or intensity of craving for chocolate (Rodin et al., 1991), and premenstrual administration of progesterone was not found to be effective in reducing cravings (Michener et al., 1999). A relatively high prevalence of women who continue to crave chocolate after menopause provides additional evidence against a significant causal role of hormonal fluctuations in craving etiology (Hormes and Rozin, 2009).

There is a lack of literature examining direct links between fluctuations in hormones and craving frequency and intensity in pregnancy; however, hormones have been implicated in prenatal craving etiology in more indirect ways. Pregnancy significantly alters sensory perception, possibly due to changes in secretion of hormones (Kuga et al., 2002; Nordin et al., 2004). When consuming plant products we ingest so-called “secondary compounds” that serve to fend off the plants’ biotic enemies and, coincidentally, give them their distinctive and flavorful aroma. In small quantities these secondary compounds have little adverse – and potentially even beneficial – effects; however, consumed in large quantities they can be allergens, mutagens, carcinogens, teratogens, and abortifacients. Expectant mothers and fetuses may be especially susceptible to these harmful effects and it has been speculated that an increase in taste and olfactory sensitivity may serve to discourage consumption of potentially toxic foods in pregnancy (Nordin et al., 2004), and could also be responsible for changing food preferences and patterns of consumption.

As many as 26% of pregnant women report altered taste sensitivity (Nordin et al., 2004), and changes in olfactory perception were reported by 65.4% of pregnant women surveyed in one study, with 75% of these women adjusting their dietary habits due to their increased sensitivity to odors (Cantoni et al., 1999). In our pilot study 18.7% of blog posts (*n* = 37) mentioned cravings for foods that were disliked prior to pregnancy. Conversely, a vast majority of pregnant women quit drinking coffee due to a unique aversion to its taste (Lawson et al., 2004), possibly driven by an increase in bitter sensitivity (Nordin et al., 2004). Parallels in changes in taste perception and the trajectory of increasing craving intensity during the first trimester (Kuga et al., 2002) constitute preliminary evidence in favor of a connection between changing sensory perception and food cravings (and, possibly, aversions) in pregnancy, however the exact nature of this link remains to be elucidated. More research is also needed to link known fluctuations in levels of hormones across gestation with reported food cravings.

## HYPOTHESIS 2: CRAVING IS A RESPONSE TO NUTRITIONAL DEFICITS

It has been speculated that perimenstrual chocolate craving is caused by a deficiency in certain nutrients that arises around the onset of menstruation and is alleviated by ingredients in the craved food. While it is possible that menstruation causes certain nutritional deficits such as low levels of iron due to blood loss (Harvey et al., 2005), it is unlikely that chocolate would serve to alleviate these needs more effectively than a variety of other foods that are not commonly craved perimenstrually (e.g., foods like red meat, egg yolks, or dark leafy greens, which provide large amounts of iron), effectively ruling out a causal role of nutritional deficits in the emergence of perimenstrual chocolate craving (Pelchat and Schaeffer, 2000; Hormes, 2014).

Fetal demands can double requirements for certain nutrients, and proper nutrition during pregnancy is critical in ensuring healthy fetal development (King, 2000). For example, a lack of adequate intake of iron can result in iron deficiency anemia and inadequate placental and fetal growth (Allen, 2000; Kaiser et al., 2008). Nutritional guidelines for pregnancy tend to highlight the importance of ensuring sufficient intake of a range of micronutrients, including iron, folic acid, B vitamins, zinc, magnesium, iodine, vitamin A, and calcium (Kaiser et al., 2002). It has been speculated that food cravings serve to prevent or alleviate nutritional deficits (or, perhaps, simply encourage the expectant mother to consume foods that provide added energy). This “nutritional deficits” hypothesis, which views craving as a mechanism to ensure adequate and balanced nutrition in pregnancy (Tierson et al., 1985), would predict that pregnant women primarily report urges for foods high in levels of micronutrients that are lacking and/or of particular importance during gestation. Such foods include dark leafy greens, which contain high levels of B vitamins, iron, magnesium and vitamin A, legumes, such as beans and lentils, which are a good source of folate, iron, and magnesium, as well as whole unrefined grains, which contain B vitamins and magnesium (Kaiser et al., 2002). Additionally, because the nutritional needs of the fetus increase as development progresses, the intensity of cravings should follow the same rising trajectory (Tierson et al., 1985).

A majority of studies found sweets, high-fat foods, and fast foods to be the predominately craved substances during pregnancy (Flaxman and Sherman, 2000; Fessler, 2002; Kaiser et al., 2002). Data from our pilot study of online posts about cravings in pregnancy suggests that while some women crave potentially beneficial proteins, fruits, or vegetables, many of the most commonly reported cravings are for high-calorie, sugary, and fatty foods (see **Table 1**). This data is largely consistent with previous studies examining cravings in a college population that found cravings for nutrient dense foods, such as fruits and vegetables, to be rarely reported (Weingarten and Elston, 1991). Thus, as is the case with perimenstrual chocolate craving, the foods typically desired by expectant women are unlikely to be the best source of nutrients needed in pregnancy. For example, an average serving of ice cream (1/2 cup, ∼60g, ∼230 calories) contains around 78 mg of calcium while the same serving size of tofu (∼60g, ∼90 calories) contains up to 160 mg of calcium and would thus constitute a much better source of nutrition. It should be noted that the available data appear to point to a higher prevalence of cravings for fruit in pregnant women, compared to other groups studied to date. More work is needed to systematically examine the seemingly higher prevalence and potential function of cravings for fruit specifically in pregnancy.

Prior studies found no evidence for a significant association between food cravings and dietary quality in pregnancy (Worthington-Roberts et al., 1989) and interestingly, potentially beneficial foods like meat and other high-protein foods appear to be among the most common aversions in pregnancy (Hook, 1978; Pope et al., 1992; King, 2000; Bayley et al., 2002). Research also suggest that the typical dietary intakes in middle- to upper-income pregnant women in the U.S. are likely to meet all dietary needs to the point where the widespread practice of prescribing prenatal vitamin supplements may lead to excessive nutrient intakes (Turner et al., 2003). Taken together, findings therefore do not support a nutritional deficits hypothesis for food cravings in pregnancy. Similarly, food cravings are unlikely to be due to a need for a general increase in caloric intake since they tend to emerge in the first half of gestation, long before a majority of fetal growth (and thus fetal demand for nutrients) takes place (King, 2000).

## HYPOTHESIS 3: CRAVING IS DUE TO PHARMACOLOGICALLY ACTIVE INGREDIENTS IN THE DESIRED FOODS

Potentially pharmacologically active ingredients in chocolate have been implicated in perimenstrual craving etiology due to their hypothesized reinforcing effects or ability to alleviate physical – and perhaps psychological – symptoms associated with menstruation, such as fatigue, irritability, bloating, or cramps (Bruinsma and Taren, 1999). The methylxanthines, a group of compounds with potentially energizing effects, are one example of a hypothesized active ingredient in chocolate (Rogers and Smit, 2000; Smit et al., 2004). However, methylxanthines are not present in large enough quantities in a normal serving size of chocolate to have a noticeable effect on anyone but the most sensitive individuals (Mumford et al., 1994; Hormes, 2014): a 60 g serving of milk chocolate contains only around 12 mg of caffeine, which is far less than the amount found in a serving of coffee, and substantially below the reliable placebo-discriminable dose (Shivley and Tarka, 1984; Michener and Rozin, 1994; Mumford et al., 1994). Other potentially active ingredients are present in even smaller quantities in the amount of chocolate typically consumed in one sitting, making it highly unlikely that their effects would be causally involved in the emergence of cravings (Rogers and Smit, 2000). The study that perhaps most compellingly demonstrates that pharmacologically active ingredients do not play a key role in the satisfaction of cravings for chocolate (during the perimenstrum or otherwise) found that white chocolate (which contains none of the pharmacologically active ingredients of milk or dark chocolate, with the possible exception of the fat-soluble cannabinoid anandamide) is far more effective at alleviating cravings than capsulated cocoa powder, which contains all of the pharmacologically active ingredients of milk and dark chocolate, but in isolation of its oro-sensory properties (Michener and Rozin, 1994).

As is the case with the perimenstrum, a variety of unpleasant symptoms like aversions to specific foods, nausea, and vomiting are widely considered hallmarks of pregnancy and it has been theorized that food cravings serve to encourage intake of substances that may help alleviate these symptoms. Prevalence estimates are somewhat varied, but it appears that between 54 and 85% of expectant women report dislike of at least one specific food, 60–80% feel nausea, and around 55% experience vomiting (Tierson et al., 1985; Bayley et al., 2002). Pregnant women tend to identify a connection between food aversions and nausea and vomiting (Schwab and Axelson, 1984; Finley et al., 1985), a link that appears contingent on principles of classical conditioning (Bernstein, 1991), suggesting that a learned taste aversion may be a possible mechanism underlying the development of specific food avoidances in pregnancy (Bayley et al., 2002). In Pavlovian terms, the avoided food acts as the conditioned stimulus, while the nausea and/or vomiting acts as the unconditioned stimulus. Findings regarding demographic variables such as age or parity that may be predictive of a greater likelihood of morning sickness^2^ have been largely inconclusive (Bayley et al., 2002).

It has been suggested that prenatal food aversions may serve the adaptive function of protecting the mother and fetus from foodborne illness. Indeed, nausea and vomiting have been associated with positive pregnancy outcomes, including lower risk of miscarriage and preterm or stillbirth (Sherman and Flaxman, 2002; Czeizel and Puho, 2004; Weigel et al., 2011). The “functional adaptation” hypothesis (largely synonymous with the Hook-Profet “maternal and embryo protection hypothesis”) proposes that nausea and vomiting are a way for women to expel and learn to avoid food-borne teratogens and abortifacients, including certain toxins found in vegetables and beverages (Hook, 1980; Profet, 1992; Flaxman and Sherman, 2000; Bayley et al., 2002; Fessler, 2002; Fessler et al., 2005). The view of nausea and vomiting in pregnancy as a protective mechanism is supported by research showing that the common aversions in pregnancy are to foods high in levels of potentially teratogenic or abortifacient agents, such as bitter vegetables, eggs, meats, and dairy products (Profet, 1992; Fessler, 2002). In addition, the most pronounced periods of nausea coincide with peak vulnerability of the developing fetus to outside toxins (Profet, 1992; Flaxman and Sherman, 2000). However, emerging discrepancies between predictions of the functional adaptation hypothesis and the available research data have led some to suggest that this account is overly simplistic and insufficient in explaining food aversions in pregnancy (Weigel et al., 2011).

In light of the high prevalence of nausea and vomiting in pregnancy it has been speculated that cravings may have developed as a way to encourage consumption of foods known to prevent or alleviate these symptoms (Bayley et al., 2002). This view parallels the theorized “medicinal” effects of ingredients in chocolate in lessening perimenstrual symptoms and is supported to some extent by the fact that food aversions and cravings frequently co-occur (Bayley et al., 2002), with some indication that aversions precede the development of cravings (Tierson et al., 1985; Bayley et al., 2002). There is also evidence of a positive correlation between the occurrence of pregnancy sickness and the development of food cravings (Whitehead et al., 1992). It should be noted that the exact nature of a possible causal relationship between food aversions and cravings may be difficult to determine due to the fact that cravings for foods providing relief from nausea may not develop for up to 2 weeks after the initial onset of the illness (Bayley et al., 2002). More research is needed to assess the temporal dynamics in the relationship between food aversions and cravings and the hypothesized role of craved foods in alleviating prenatal nausea and vomiting.

## HYPOTHESIS 4: CRAVING IS CAUSED BY CULTURAL AND PSYCHOSOCIAL FACTORS

Culture has long been known to be a powerful determinant of eating behaviors and our proposed model of craving etiology hypothesizes a key role of cultural norms in the emergence of food cravings (**Figure 1**). In the absence of strong evidence in favor of physiological and biochemical causes of perimenstrual chocolate craving, studies have consistently identified significant differences in the overall prevalence, types, and gender ratio of food cravings across various cultures. For example, while chocolate is by far the most commonly craved food in the U.S., hardly anyone in Egypt endorses strong urges for chocolate or general sweet cravings (Parker et al., 2003). Rice is the most widely craved food among women in Japan (Komatsu, 2008), a finding that highlights the strong influence of culture and culinary tradition on food-related preferences. As noted previously, American women are about twice as likely as U.S. men to crave chocolate (91 versus 59%), but men and women in Spain are almost equally likely to report strong urges for chocolate (90 and 78%, respectively; Osman and Sobal, 2006). The word “craving” does not translate into most languages outside of English, suggesting that the construct may be less important or altogether unknown in other cultures (Hormes and Rozin, 2010). Taken together these findings support the view that culture plays a central role in the emergence of perimenstrual chocolate craving and highlight the importance of studying the role of contextual and psychosocial factors in the etiology of cravings in other domains.

Conflicting attitudes toward foods like chocolate that are perceived to be simultaneously appealing and “forbidden” have recently been hypothesized to be associated with a greater likelihood of craving (Cartwright and Stritzke, 2008; Hormes and Rozin, 2011). Ambivalent feelings toward chocolate and similar foods are likely to be especially common in U.S. women who are exposed to a culture that promotes largely unrealistic ideals of female beauty (Thompson and Stice, 2001), while at the same time providing easy access to large quantities of highly palatable and calorically dense foods in what has been termed an “obesogenic” environment (Swinburn et al., 1999). Evidence suggests that efforts to avoid foods that cause these conflicting feelings may have the paradoxical effect of increasing the likelihood of craving. The result is a sort of “vicious cycle” of alternating restraint and overconsumption or binge eating. Multiple studies have demonstrated that dieting to lose weight and restricting intake of well-liked foods are associated with an increase in the salience of (internal and external) cues related to that food and, as a result, in the frequency of cravings (Placanica et al., 2002; Hill, 2007; Smeets et al., 2009; Kemps and Tiggemann, 2009; Hollitt et al., 2010; Durkin et al., 2012; Massey and Hill, 2012). A recent study found that U.S. women who experience cyclical increases in chocolate craving report significantly greater levels of dietary restraint, less flexible control over food intake, more guilt when consuming chocolate, and higher body mass indices (Hormes and Timko, 2011), supporting the notion that eating-related pathology may play a causal role in craving etiology.

Given these findings it seems warranted to examine cultural differences related to food cravings in pregnancy, and to try and identify contextual and psychosocial factors involved in their emergence. Evidence in favor of cross-cultural differences in craving prevalence and a causal role of psychosocial factors such as conflicting attitudes toward highly palatable foods, eating disorder symptoms, and dietary restraint would support the applicability of the proposed model in understanding food cravings in pregnancy. There is a small body of literature examining the prevalence, types, and correlates of prenatal food cravings and aversions as well as rates of nausea, vomiting, and pica in pregnancy in countries outside the U.S. Evidence suggests that all these phenomena exist in diverse cultures. For example, in a sample of 99 pregnant British women 61% reported experiencing strong urges for specific foods (Bayley et al., 2002). Between 64.9 and 79% of pregnant women in Tanzania have been found to experience food cravings, with craving intensity peaking in the first trimester (Nyaruhucha, 2009; Patil, 2012; Steinmetz et al., 2012). In India the term *dola-duka* is used to describe the experience of food aversions and cravings in pregnant women (Obeyesekere, 1963). *Duka* refers to the period in which a woman experiences nausea, vomiting, and weakness. *Dola* appears synonymous with what U.S. culture would deem a *craving* and refers to the desire to obtain a substance for consumption.

Prenatal cravings thus seem to exist outside the U.S. and prevalence appears fairly stable across the different countries surveyed to date. However, data also suggest that there are culture-specific differences in reported types, perceived meaning, and psychosocial correlates of cravings in pregnancy. An early study found that pregnant Indo-Ceylon women experience nausea, vomiting, and aversions associated with foods reflective of their traditional role as a wife and mother (e.g., time and effort spent preparing traditional foods like rice, everyday curries, tortillas from millet, and jiggery; Obeyesekere, 1963). Cravings reported by these women were categorized as childhood foods (e.g., sweets), foods expressing hostility, rare or expensive foods, festival foods, sour foods, male or phallic foods, and idiosyncratic foods (i.e., those that have personal significant meaning to the individual; Obeyesekere, 1963). Pregnant women in Nigeria proclaimed that their most craved foods (fruits, vegetables, and cereals) provide nutritional benefits, justifying their consumption with the belief that they are a good source of body building nutrients, serve as a mild laxative, and are easy to prepare (Olusanya and Ogundipe, 2009). The most common cravings reported by a sample of 545 Tanzanian pregnant women (i.e., reported by more than 25% of cravers) were meat and fish, vegetables, fruits, and grains (Patil, 2012). Provision of craved foods to pregnant women by their husband and his family is considered an expression of social support in rural Tanzania (Patil, 2012). In Indo-Ceylon cultures, the *dola* cannot be satisfied until the substance is consumed, and if it is not satisfied the woman is said to endure significant anxiety and stress until the compulsion is relieved (Obeyesekere, 1963). These data provide preliminary support in favor of a role of cultural associations in the types of food cravings in pregnancy, though more work is needed to systematically compare and contrast the nature, prevalence, and significance of food cravings in pregnancy across diverse cultures.

As noted previously, it has been speculated that food cravings may be a risk factor for excess weight gain in pregnancy. However, interesting cultural differences in the prevalence of excess weight gain in pregnancy suggest that a link between cravings and GWG may be unique to the U.S. (or perhaps North America)^3^: compared to more than 50% of U.S. women gaining too much weight prenatally, only 14.5% of obese and 30.4% of normal-weight women in Sweden were found to gain more than 16 kg (35.3 lbs) during singleton term pregnancies (Cedergren, 2006). Just over 20% of German mothers reported GWG of more than 17 kg (37.5 lbs; von Kries et al., 2011). In a prospective study of pregnant Vietnamese women, only 19.6% gained between 15 and 20 kg (33.1–44.1 lbs), and a mere 2.7% experienced GWG exceeding 20 kg (44.1 lbs; Ota et al., 2011). Based on these data it can be speculated that some factor that is unique to U.S. culture increases the likelihood that women gain excess weight in pregnancy. This hypothesis is supported by the finding that weight gain in pregnancy appears to be tied to a woman’s level of acculturation to U.S. culture: Hispanic women who spent <10 years living in the United Sates were 50% less likely to gain above the threshold for GWG recommended by the IOM compared to third generation women (Chasan-Taber et al., 2008). Level of acculturation to U.S. culture in Hispanic women was also found to be a determinant of the types of foods consumed during pregnancy such that the less acculturated women reported consuming primarily traditional foods (Tovar et al., 2010).

A feeling of ambivalence toward highly palatable and calorically dense foods is a central aspect of the proposed model of craving etiology. It is thought that these ambivalent feelings heighten the salience of food-related cues, resulting in an increased likelihood of craving and subsequent consumption of the desired food (Hormes, 2014). There is some evidence for conflicting feelings related to food in pregnant women. For example, it has been argued that in U.S. women, the idea of “eating for two” takes on moral significance such that healthful eating in pregnancy is consistent with the perceived ideal of a good mother, while consumption of unhealthy foods is the cause of a considerably conflicting feeling (Copelton, 2007). Similarly, a survey of pregnant women with gestational diabetes in Canada found that cravings were frequently perceived to be specifically for “forbidden” foods, such as sweets (Hui et al., 2014).

Menstrual cravings have previously been found to be associated with elevated levels of eating disorder symptomology (Hormes and Timko, 2011), and it can be hypothesized that maladaptive eating-related attitudes and behaviors may also increase the likelihood of prenatal cravings. It has been suggested that the presence of disordered eating behaviors could specifically heighten the risk of overconsumption in response to external and internal food-related cues in pregnancy (Fairburn and Welch, 1990; Abraham et al., 1994; Clark and Ogden, 1999). The prevalence of eating disorders in pregnant women (1%) is generally estimated to be equal to or perhaps even lower than that in the general population (1–3.5%; Hudson et al., 2007). In fact, a majority of women experience a decrease in dietary restraint and an increase in energy intake, weight, and overall body satisfaction during pregnancy (Fairburn and Welch, 1990; Wiles, 1993; Clark and Ogden, 1999). In a number of studies it has been found that for individuals diagnosed with bulimia nervosa, episodes of bingeing and purging decreased during pregnancy (Crow et al., 2004). However, for women with a history of problematic eating, pregnancy can trigger an increase in weight concern, sensitivity about body shape, and even maladaptive eating-related behaviors like bingeing and purging (Clark and Ogden, 1999). An early study suggests that both food cravings and aversions may be especially common in women who were particularly “picky” or had high levels of food faddiness as children, as well as those who endorse stress-induced appetite changes (Dickens and Trethowan, 1971). Women with a lower pre-pregnancy body mass index (BMI) and a history of disordered eating appear at greater risk to exceed guidelines for recommended GWG (Chu et al., 2009; Laraia et al., 2009). Pregnant women with a recent or past eating disorder were also found to be more likely to abuse laxatives, to engage in self-induce vomiting, and to exercise as compared to normal controls (Micali et al., 2007).

Episodes of binge eating are the most frequent disordered eating behavior in pregnant women (Fairburn and Welch, 1990; Abraham et al., 1994; Soares et al., 2009). The frequency of binge eating during pregnancy has significant effects on the mother’s health, particularly regarding GWG (Soares et al., 2009). Caucasian women deemed restrained eaters (i.e., those who frequently think about their diet and weight and make attempts to restrict their dietary intake) are significantly more likely than unrestrained eaters to exceed guidelines for recommended GWG (Conway et al., 1999), a finding that supports the hypothesis that pregnancy acts as a time for women to legitimize seemingly excessive food intake, disregarding any previous attitudes and intentions to eat less (Clark and Ogden, 1999). Similar to restrained eaters, dieters^4^ have also been found to endorse more episodes of overeating during pregnancy, compared to non-dieters (Fairburn and Welch, 1990). There are two possible explanations for this finding: women either (a) began dieting in response to already having gained excess weight, or (b) abandoned prior dieting attempts while pregnant and engaged in disinhibited eating, resulting in excess weight gain (Fairburn and Welch, 1990). Interestingly, for a sample of African–American women levels of restraint were relatively low and were not found to be predictors of excess GWG (Allison et al., 2012), suggesting that restraint may be more prevalent in certain cultures and ethnicities, and as a result have a different effect on GWG. The notion that pregnancy constitutes a culturally sanctioned excuse for dieters and women high in dietary restraint (and potentially eating disorder symptoms) to consume (and possibly overconsume) highly palatable foods that are otherwise perceived as taboo due to their high caloric content is consistent with the proposed model. Interestingly, the idea that pregnancy is a time when one does not need to feel accountable for one’s food intake, (i.e., a time of disinhibition), has been found to be most commonly endorsed by women classified as habitual dieters prior to pregnancy (Fairburn et al., 1992; Clark and Ogden, 1999; Mumford et al., 2008). Of note, it has been suggested that continuous pregravid dieting may affect the women’s ability to accurately distinguish hunger and satiety cues, which may contribute to excess energy intake in pregnancy (Mela and Rogers, 1998; Conway et al., 1999; Mumford et al., 2008).

Additional support for the view of pregnancy as a socially acceptable time for women to indulge comes from sociological research that finds that pregnant women take on a more functional view of their body, which legitimizes divergence from cultural ideals of thinness and restraint (Bailey, 2001; Dworkin and Wachs, 2004). In our qualitative pilot study, among the women posting about their cravings on the pregnancy-related blogs, negative affect related to cravings was rare and only mentioned by 6.1% (*n* = 12) of respondents. This low number may be due in part to the fact that the nature of the message board encouraged reports of cravings, but it may also reflect a more general sense that cravings in pregnancy are acceptable or maybe even enjoyable. Remarkably, only 4.5% (*n* = 9) of respondents described efforts to resist their cravings. These figures stand in stark contrast to the high levels of negative affect and conflicting approach-avoidance tendencies typically found to be associated with craving in the general population (Macdiarmid and Hetherington, 1995; Cartwright and Stritzke, 2008; Hormes and Rozin, 2011).

It thus appears that in the U.S., culture-specific norms, beliefs, and customs may allow or even encourage prenatal cravings and intake of foods that may otherwise be considered “taboo” (Snow and Johnson, 1978). As a result these views on cravings may leave pregnant women susceptible to overconsuming high calorie foods, resulting in excess weight gain, especially for women high in restraint and those with pre-existing eating disorder symptoms.

## CONCLUSION AND DIRECTIONS FOR FUTURE RESEARCH

While some have argued that the mechanisms underlying food cravings in pregnancy differ from cravings experienced at other times (Gendall et al., 1997), we believe that the evidence presented here strongly supports the assumption that our proposed model of craving etiology applies to cravings in both the perimenstrum and pregnancy. We have reviewed evidence in favor of and against four major hypotheses regarding the etiology of perimenstrual and pregnancy cravings, implicating hormones, nutritional deficits, rewarding or reinforcing ingredients in the craved foods, and a complex set of cultural and psychosocial variables. Regarding perimenstrual chocolate cravings, evidence in favor of physiological/biochemical causes has been sparse. The literature on eating behaviors in pregnancy is largely consistent with these findings insofar as hormonally driven changes in sensory perception and the effects of potentially active ingredients in craved foods seem unlikely to be causally involved in the emergence of prenatal cravings. Prior research on the role of cultural and psychosocial factors in the etiology of food cravings in pregnancy is somewhat limited; however, existing studies point toward interesting cultural similarities in craving prevalence, as well as noteworthy differences in craving types and correlates that are consistent with the assumption that culture plays a key role in bringing about cravings in pregnancy. Furthermore, the observed link between food cravings and excess GWG gain may be unique to women in the U.S. or North America.

Factors influencing food cravings and weight gain in pregnancy are complex (Paul et al., 2013), and there are several important limitations inherent in existing research that must be addressed in future studies. For example, cross-cultural differences in prevalence of pregnancy cravings and GWG may simply be reflective of differences in the availability of and access to certain foods. The sample of Tanzanian pregnant women surveyed by one of the study referenced earlier was described as “marginally nourished,” with food insecurity and hunger among the most common stressors faced by this group (Patil, 2012). Many of the key studies examining food cravings and aversions in pregnancy are somewhat dated. Assuming a key role of culture in craving etiology and given the fact that cultural norms and practices can change significantly over the course of even just a few decades it will be important to replicate some of the key studies cited here to determine if findings hold in current samples of pregnant women.

In addition to addressing these limitations we propose that future research in this field should focus on four specific areas of investigation: (1) validation of existing measures assessing food cravings and related behaviors and attitudes specifically in pregnant women, (2) real-time assessment of food cravings using ecological momentary assessment (EMA), (3) longitudinal tracking of eating disorder symptoms, food cravings, and GWG to determine causality, and (4) identification of targets for interventions to increase proper nutrition and decrease the risk of excess weight gain in pregnancy.

The failure of the term “craving” to lexicalize in most languages outside of English impacts the extent to which studies can accurately assess cross-cultural differences in the nature of food cravings in pregnancy (Hormes and Rozin, 2010). More work is needed to determine equivalence of terminology used by women in other countries to describe strong urges for specific foods. For instance, in one study a significant portion of the Hispanic women surveyed reported wanting to “eat junk food,” and it can be speculated that these reports may be comparable to accounts of cravings in North American women (Tovar et al., 2010). There is also a lack of measures of food cravings and related attitudes and behaviors that have been validated specifically in pregnant women. Future studies should focus on determining the psychometric properties of key measures typically used in research on food cravings specifically in women in pregnancy^5^. Comparable efforts have previously been exerted in order to validate measures of anxiety specifically in the perinatal period (Meades and Ayers, 2011).

Many prior studies of food cravings in general, and specifically in pregnancy, are retrospective in nature (Nordin et al., 2004). Given the transient nature of the craving experience it is unlikely that craving episodes are accurately remembered following extended delays. Real-time neural correlates of food cravings are beginning to be examined using different forms of magnetic imaging (Pelchat et al., 2004; Frankort et al., 2014); however, this approach is not feasible in studying cravings in pregnancy due to the adverse effects of performing magnetic imaging on the health of the fetus. An area of research that has been receiving increasing attention and that is appropriate for the real time assessment of cravings in pregnant women is the use of EMA. For example, EMA has recently been utilized in studies of temptation and lapses in dieting (Carels et al., 2001), as well as cravings associated with smoking cessation (Waters et al., 2014), marijuana use (Buckner et al., 2011), and detoxification from substance use (Marhe et al., 2013). Real-time assessment of food cravings has also been used to examine the association between exposure to food cues in the external environments and craving and subsequent consumption in adolescents (Grenard et al., 2013). Compared to paper and pencil methods (i.e., those that provide the participant with the paper and pencil measures and cue them in advance to fill out the questionnaires at specific times throughout the day) electronic EMA (i.e., completion of measures in real time using an electronic device) was found to have a higher response rate when tracking food cravings and food intake (Berkman et al., 2014). Berkman et al. (2014) aimed to identify whether certain individual characteristics (i.e., BMI) increased or decreased responses using EMA technology. Findings showed that individuals with greater body mass indices were less likely to respond in the paper and pencil method as compared to the electronic EMA method. Furthermore, higher BMI was negatively correlated with latency response time in both groups. To the best of our knowledge, EMA has not yet been used to assess food cravings in real time in pregnant women. Thus, it is suggested that future research aim to implement the use of this technology to gage the intensity, frequency, types, and temporal patterns of food cravings specifically in this population.

The impact of GWG on maternal and child health has been deemed to be of great public health importance (Kaiser et al., 2009), and research to identify social, cultural, and environmental risk factors for excess GWG has been called for by the IOM (Rasmussen and Yaktine, 2009). Much of the work in this area has been cross-sectional in nature and there is an urgent need for longitudinal studies in order to determine with certainty the nature of the hypothesized associations between psychosocial risk factors, cultural variables, food cravings and consumption, and weight gain in pregnancy (i.e., do cravers gain more weight in pregnancy than non-cravers, and if so, what are the causes of cravings?; Hook, 1978; Bayley et al., 2002). The ultimate goal of research in this area is to identify predictors of overweight and obesity in pregnant women in order to develop interventions that encourage good nutrition and healthy weight gain. Prior research on interventions targeting eating behaviors has had somewhat mixed results. Some studies implementing behavioral interventions for weight gain during pregnancy found that programs had a significant effect only on mothers with low socioeconomic status (Olson et al., 2004). However, there is also reason for optimism. For example, the Fit for Delivery program invites pregnant women to complete a face-to-face visit where guidelines for appropriate weight gain and behaviors related to proper nutrition are discussed. In one randomized controlled trial women (*n* = 201) were assigned to the intervention, which started between 10 and 16 weeks gestation. Following the face-to-face visit, women in the experimental group received postcards encouraging the continuation of healthy behaviors as well as (a minimum of) three phone calls from a dietician over the course of their pregnancy. Findings showed that the intervention reduced excessive GWG for normal weight women, as well as increased the likelihood that pregravid normal or over-weight women returned to their pre-pregnancy weight by six months postpartum (Phelan et al., 2011).

Thus, there is preliminary evidence in support of the effectiveness of behavioral interventions targeting weight and eating behaviors in pregnancy; nevertheless, research in this area remains lacking. It has been suggested that going forward, an emphasis on the prevention (as opposed to the treatment) of weight-related problems in pregnancy may be key (Cox and Phelan, 2008). We hope that the present discussion outlines avenues for identifying novel targets for future intervention programs. The adverse effect of excess weight gain in pregnancy on weight-related pathology across the lifespan is well documented. Importantly, however, it has also been shown that pregnancy is a “teachable moment,” with implementation of behavior change during this time frequently resulting in especially long-lasting positive impact due to the mother’s enhanced awareness of the effects of her behaviors on the health of the fetus (Phelan, 2010; Phelan et al., 2011). Targeting eating attitudes and behaviors in pregnant women may thus provide a unique opportunity to improve mothers’ and children’s weight-related health over the long term.

## Conflict of Interest Statement

The authors declare that the research was conducted in the absence of any commercial or financial relationships that could be construed as a potential conflict of interest.

## Footnotes

1 This study sought to gather information on the types of food cravings currently reported by pregnant women in the U.S. by surveying women’s posts on the topic of food cravings on two popular pregnancy-related websites: www.thebump.com and www.whattoexpect.com. Data were collected in the fall of 2012. We searched the “community” forums on the two websites for the term “craving” and examined responses contained in the first 20 message threads generated by that search, resulting in a sample of 200 unique posts. We excluded search results that were unrelated to food cravings, as well as posts that prompted women to comment on specific types of cravings (e.g., “Is anyone else craving lemonade?”) so as not to skew our results in favor of any one type of food or beverage. We categorized responses according to specific categories (e.g., fast food, prepared dishes, pre-packaged foods) and types of foods (e.g., fruits, vegetables, sweets), as well as certain flavor profiles (e.g., sour, sweet, salty). We also coded responses noting cravings for beverages or non-food substances. In addition, we looked for mentions of specific hypotheses regarding the perceived causes of cravings, efforts to resist cravings, negative affect related to cravings, or the notion that pregnancy may serve as an excuse to consume otherwise forbidden foods.

2 It should be noted that the term “morning sickness” is somewhat of a misnomer, with only about 17% of women who felt nausea and 31% of women who experienced vomiting during pregnancy reporting that symptoms occurred exclusively in the morning (Whitehead et al., 1992).

3 Of note, there are marked differences in guidelines for GWG in different countries. In a review comparing national GWG and energy intake recommendations (EIR), 13 of 22 countries surveyed had guidelines similar to those put forth by the IOM and adopted by the U.S., Canada, Finland, Italy, parts of Australia, and parts of Asia (specifically, Vietnam and Singapore). All either used the 2009 IOM or very comparable guidelines (Alavi et al., 2013). Parts of Western Europe recommend GWG in the lower end of the IOM suggestions (10–15 kg or 22–33 lbs). In India and Africa (8–10 kg or 17.6–22 lbs), the Phillipines (11–12.5 kg or 24.3–27.6 lbs), and Chile (12–13 kg or 26.5–28.7 lbs), official guidelines all suggest significantly lower weight gains for a normal weight expectant mother, compared to the thresholds recommended by the IOM (Alavi et al., 2013).

4 Individuals were classified as “dieters” if they gave a clear history of dieting prior to pregnancy. Of those assessed, 54% reported having dieted in the past to modify their shape and/or weight (Fairburn and Welch, 1990).

5 These measures include the Eating Disorder Diagnostic Scale (Stice et al., 2000), Dutch Eating Behavior Questionnaire (Van Strien et al., 1986), the Three Factor Eating Questionnaire (Stunkard and Messick, 1985), Food Craving Questionnaire (Cepeda-Benito et al., 2001), Food Craving Inventory (White et al., 2002), general psychosocial assessments like the Depression, Anxiety, and Stress, Scale (Henry and Crawford, 2005), and others.
